# Visualizing implementation: contextual and organizational support mapping of stakeholders (COSMOS)

**DOI:** 10.1186/s43058-020-00030-8

**Published:** 2020-05-27

**Authors:** Steven L. Bernstein, June Weiss, Leslie Curry

**Affiliations:** 1 Yale Center for Implementation Science, Department of Emergency Medicine, Yale School of Medicine, New Haven, USA; 2 Department of Chronic Disease Epidemiology, Yale School of Public Health, 464 Congress Ave., Suite 260, New Haven, CT 06519 USA; 3 Department of Health Policy and Management, Yale School of PublicHealth, 464 Congress Ave., Suite 260, New Haven, CT 06519 USA

**Keywords:** Stakeholder, Stakeholder mapping, Implementation, Smoking cessation, Stakeholder analysis, Implementation science

## Abstract

**Background:**

While stakeholder mapping is common in public policy, social sciences, and business management, this tool has not often been used in healthcare settings. We developed a new method of healthcare stakeholder mapping, which we call Contextual and Organizational Support Mapping of Stakeholders (COSMOS), to identify and assess key stakeholders in an implementation project. Stakeholder mapping allows the implementation team to assess and visually display all relevant stakeholders, their support for the project, and their ability to facilitate—or hinder—project implementation.

**Methods:**

The COSMOS model was developed to visualize the stakeholders involved in a hospital-based study conducted from 2013–2016. In this study, a new screen prompt and order set were embedded in the electronic health record to facilitate the identification and treatment of adult smokers admitted to the hospital. Physicians were the unit of randomization; physician behavior and tobacco quit rates among patients were followed for 1 year. Qualitative interviews with hospital administration, physicians, and information technology (IT) personnel (*n*=24) were conducted to identify the components and characteristics of the COSMOS.

**Results:**

The COSMOS consists of an organizational chart identifying all key stakeholders, with manipulation of colors and borders of the component boxes to indicate stakeholder support for the implementation project, and degree of criticality to its success. The COSMOS visualization informed the team’s subsequent work by identifying potential impediments that might require additional attention to garner and maintain support throughout the project. In addition, the approach has proved to be a useful tool to explain these concepts to trainees in implementation science.

**Conclusion:**

The COSMOS schematic provides a visually rich means of identifying stakeholders, understanding their relationships to each other, displaying their level of support for the proposed implementation, and noting their criticality to the effort. The COSMOS can support researchers, project teams, administrators, and others engaged with implementation science-related work in healthcare, as well as other fields such as education, government, and industry.

Contributions to the literature

- Stakeholder analysis is a critical element in the planning of program implementation. Available models to visualize organizational stakeholders lack specific features that might enhance their utility.
- We introduce the COSMOS, a visual guide to stakeholder analysis that uses an organizational chart as a platform, upon which are layered additional visual elements that denote the criticality of individual stakeholders to project success, as well as their level of enthusiasm for the project. Additional contextual statements provide further actionable data for the project team.
- COSMOS visualizations may be used to characterize the full set of activities that attempt to identify, describe, and characterize all components of an environment in which a new program is to be implemented.

## Background

### Introduction

Successful implementation of a new policy, process, or practice depends on many factors, including identification and analysis of stakeholders, which may include the individuals, units, agencies, or organizations needed to implement a policy, process, or practice [1, 2]. Stakeholder analysis includes identifying these individuals and organizations, understanding their relationships to each other, their interest in implementation success, their criticality in implementation, and their unique motivators to support (or oppose) the project. Taxonomies such as the Consolidated Framework for Implementation Research (CFIR) [3] and Promoting Action on Research Implementation in Health Services (PARiHS) [4] and models such as Assess/Innovate/Develop/Engage/Devolve (AIDED) [5] acknowledge, implicitly or explicitly, the importance of stakeholder analysis in the domains of the inner setting and the individuals involved in the implementation.

For the team leading the implementation, visualization of the stakeholders, their interrelationships, levels of support for change, and criticality to implementation success can help identify individuals and organizations who are likely to facilitate, or hinder, program implementation. Several prior visualization and mapping schemes have been developed, largely outside healthcare contexts. We briefly summarize illustrative models in the next section, followed by their limitations in terms of application in healthcare settings, and finally introduce a new proposed method to visualize stakeholder mapping, which we call the COSMOS.

### Introducing the COSMOS

Recent years have seen an explosion in the biological sciences of “-omics”: genomics, proteomics, metabolomics, and related sciences [6]. These sciences address the complete set of genes, proteins, and metabolic processes that are contained within an organism. Similarly, at a larger level, we believe the same principles can be adapted to late-stage translational science to facilitate implementation projects embedded within discrete organizations like clinics, hospitals, healthcare systems and public health agencies, or even larger units like cities and countries.

The COSMOS is a visual representation—a map—of the set of all individuals, units, organizations, and agencies needed to introduce a new policy, process, or practice into a healthcare setting. The COSMOS displays the desired attributes of organizational structure and provides qualitative assessments of project support and criticality to success of each stakeholder. Certain components of the COSMOS are likely to be found in all implementation programs. They may include individuals such as practitioners (both physicians and midlevel providers), healthcare technicians, administrators, information technology (IT) specialists, public officials, and payors. They may also include the organizations in which these individuals are embedded: clinics, hospitals, practices, community health centers, public health agencies, and nonprofit organizations.

Yet, the COSMOS is highly dependent on context, and may evolve over time as the change moves from dissemination to adoption to implementation to maintenance. Because of differing organizational structures, reporting relationships, job responsibilities, informal social networks, and regulatory, fiscal, and clinical environments, different organizations implementing the same program may have different COSMOS maps. Conversely, a single organization implementing different programs may also have different COSMOS maps.

An in-depth and comprehensive knowledge of the COSMOS can support implementation success through the following: (1) identifying all relevant stakeholders in an organization or system, (2) clarifying their formal reporting relationships as applicable, (3) quantifying the relative importance of the stakeholders to project success, (4) assessing their relative support for the project, and (5) providing important contextual elements that can facilitate implementation. The comprehensive information synthesized in the COSMOS can help the project team craft a plan for implementation, assist with strategic negotiations with key stakeholders, and maximize the allocation of personnel and resources to ensure project success.

### Prior models of stakeholder mapping

Stakeholder mapping is common in the management and policy literature [1, 7]. While models offer slightly differing approaches to visualizing stakeholders, most include variations of certain common elements:

1. listing the stakeholders
2. displaying their power to facilitate or hinder the implementation
3. displaying their interest in the intervention
4. displaying their proximity to where the implementation will occur in the organization.

**Fig. 1:**
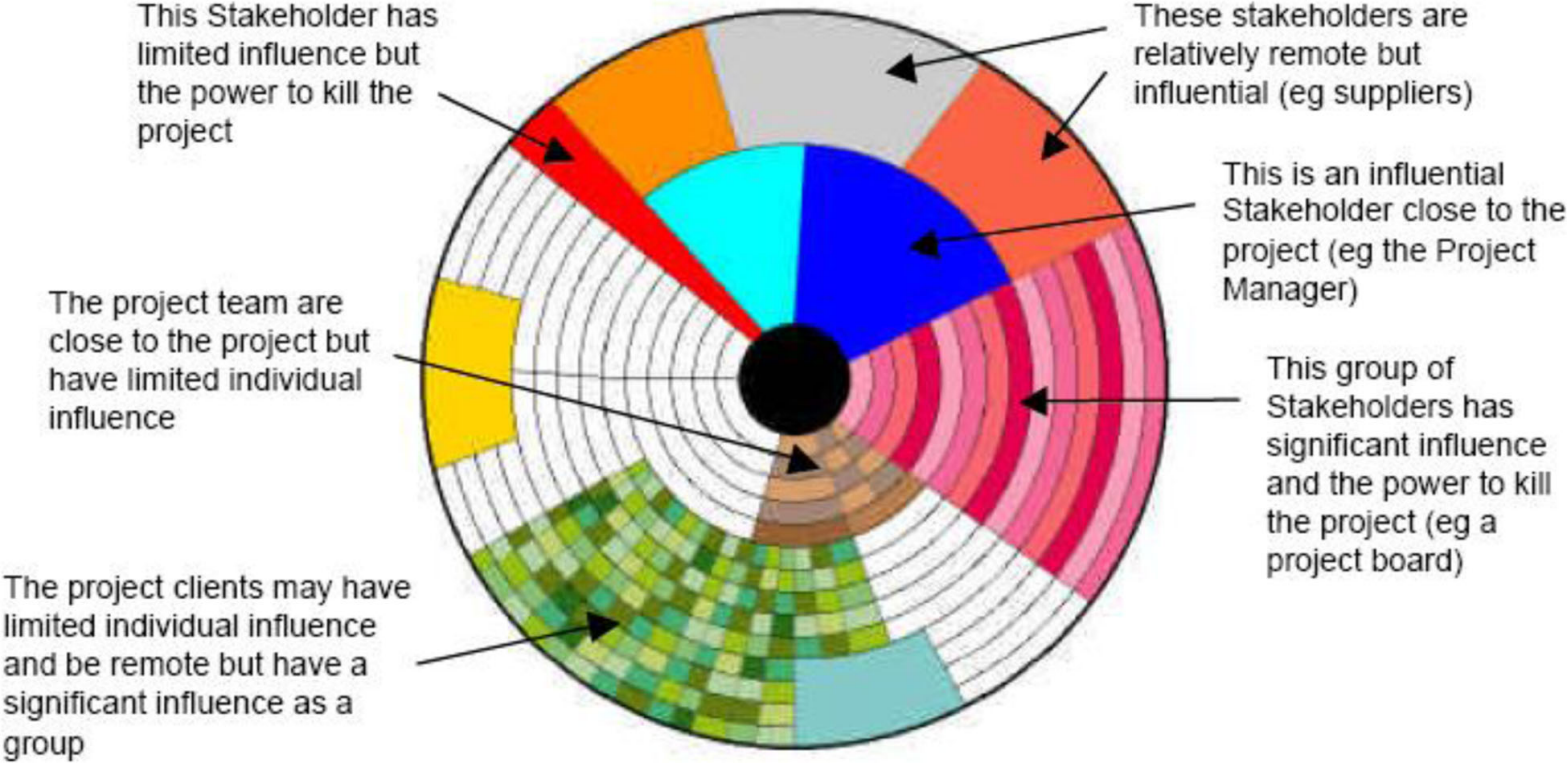
The stakeholder circle. Reproduced from Bourne 2006

The stakeholder circle of Bourne and Walker was one of the earliest models [8–10] (Fig. 1). The stakeholder circle is a stylized representation of stakeholders’ proximity to the project manager (the solid black circle at the center). Stakeholders are represented by colored wedges, with distance (or proximity) to the project manager representing the stakeholders’ closeness to the project, contact with the project manager representing the ability to assist (or block) project implementation, and the size of the wedges representing scope and scale of individual stakeholders’ influence.

**Fig. 2:**
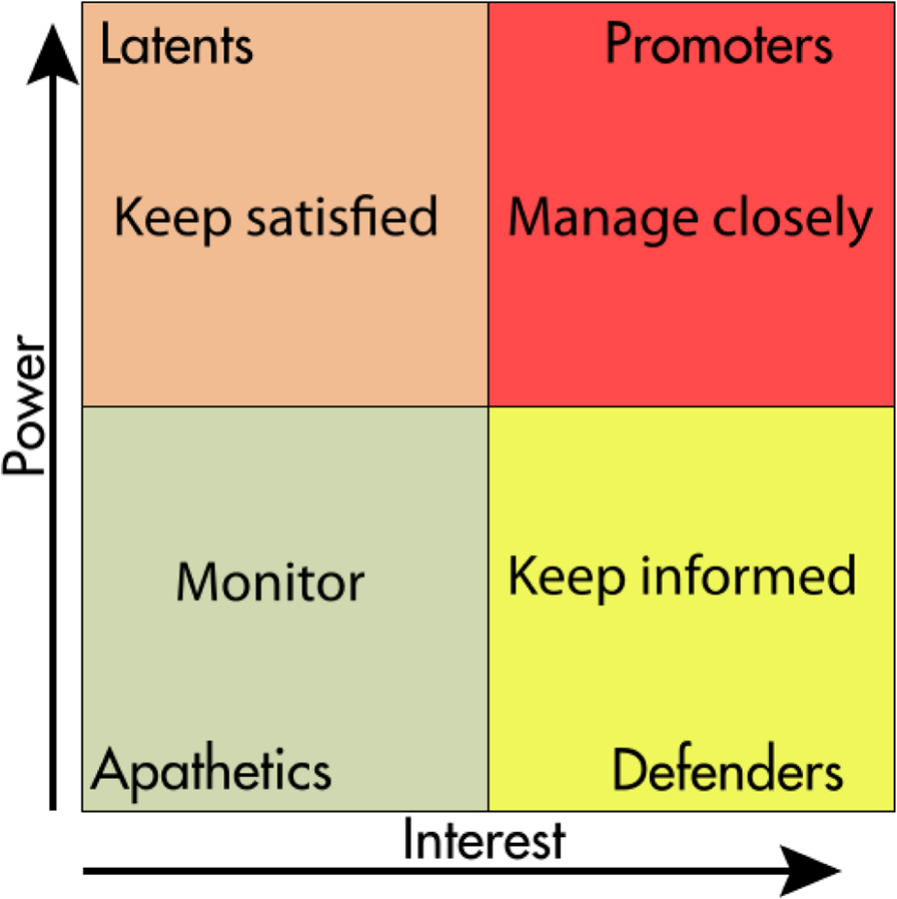
Power-interest grid of Mendelow [11]

**Fig. 3:**
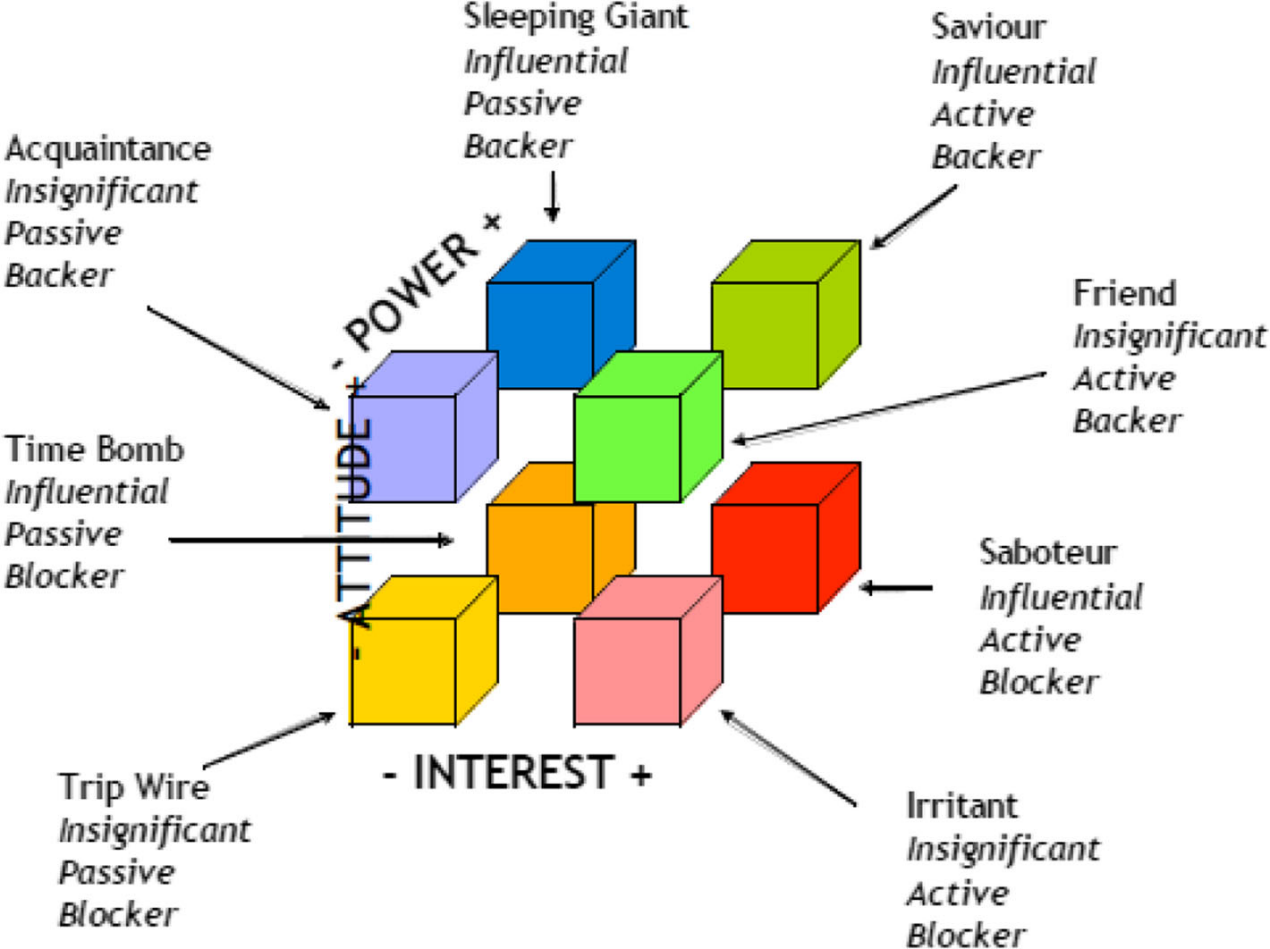
Power-interest-attitude matrix of Murray-Webster and Simon [12]

The power-interest 2 × 2 matrix of Mendelow [11] has been widely used to categorize stakeholders in four domains: high power/high interest, high power/low interest, low power/high interest, and low power/low interest (Fig. 2). Murray-Webster and Simon [12] add a third dimension, that of attitude, to the power-interest grid (Fig. 3).

**Fig. 4:**
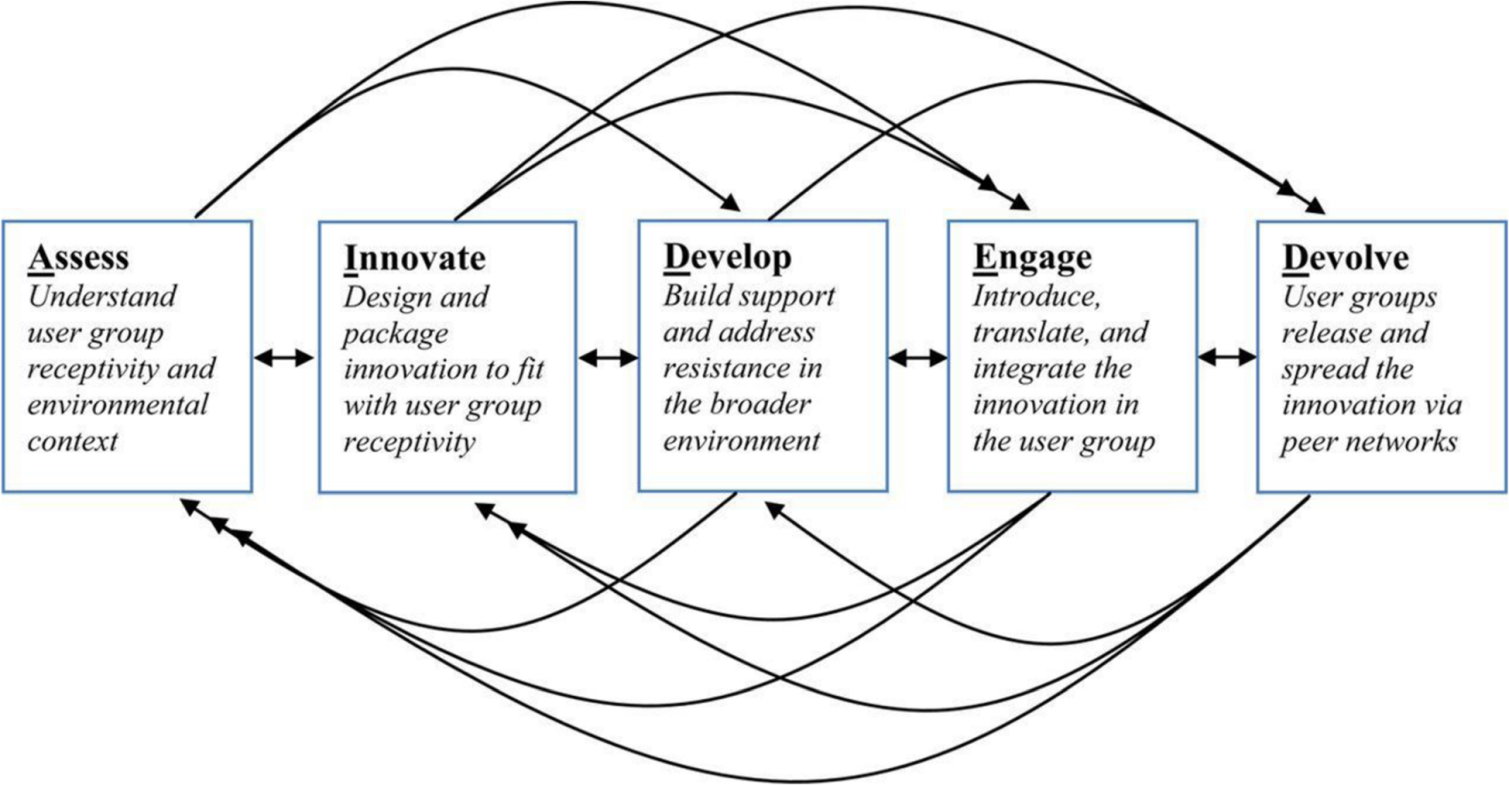
The AIDED model. From Bradley et al. [5]

Bradley et al. [5] described a model called Assess/Innovate/Develop/Engage/Devolve (AIDED), to assist in planning scale-up of interventions (Fig. 4). AIDED’s components include assessing user group receptivity and environmental context; innovating, or designing, the intervention to match user receptivity; developing support and identifying resistance to implementation; engaging with the end users; and devolving, or facilitating the users in disseminating the change across peer networks. The first component of AIDED corresponds with identifying and developing stakeholder maps. The model was used in a scale-up of innovations in family healthcare in low- and middle-income countries.

These models are useful representations in defining individuals and groups, their varying levels of interest and attitude toward the innovation, and their power to facilitate or block the implementation. In our own work, we found that each approach had certain limitations, particularly when used to guide implementation in a healthcare setting. These include the following:

1. Insufficient attention to the table of organization and the power relationships among individuals, units, and organizations*.* Healthcare organizations—medical centers, hospital systems, ambulatory care networks, public health agencies—are typically highly matrixed, with organizational charts that evince complex reporting relationships. In the current environment, where mergers of healthcare organizations are common and turnover at leadership levels may be frequent, it becomes critically important for teams tasked with implementation to have a deep, nuanced understanding of the organization, its hierarchical structure, reporting relationships, and interrelationships among disparate components of the organization. Current models and visualizations of stakeholders typically do not provide a sufficiently granular picture of the healthcare system’s organizational chart, reporting relationships, or the informal relationships that develop within organizations that can facilitate (or impede) the work of implementation.
2. A highly stylized format that may lack intuitive meaning to an implementation team, embedded in a healthcare setting that may not be fluent in the vocabulary and concepts of management. The models currently available make use of matrices, wheels, and other information-dense visualizations that, while communicating important information, may not be intuitively obvious to the team tasked with the implementation.
3. A general lack of prior development and testing in healthcare settings. Most prior models of stakeholders have addressed other types of organizations, largely in the business world, occasionally in the nonprofit sector. To the extent that large organizations share many common structural features, this may not present a great concern. However, healthcare personnel, providers, administrators, and others are likely to prefer assessing implementation strategies with models that are healthcare-specific.

## Methods

In order to address these limitations, we developed a new method to visualize stakeholders, their interrelationships, criticality to project success, and level of support for the project. A high priority for the team was to create a COSMOS map with a format that can be easily understood by healthcare providers and administrators skilled in the provision and organization of care, but perhaps less versed in the principles of management.

### Case study

**Fig. 5:**
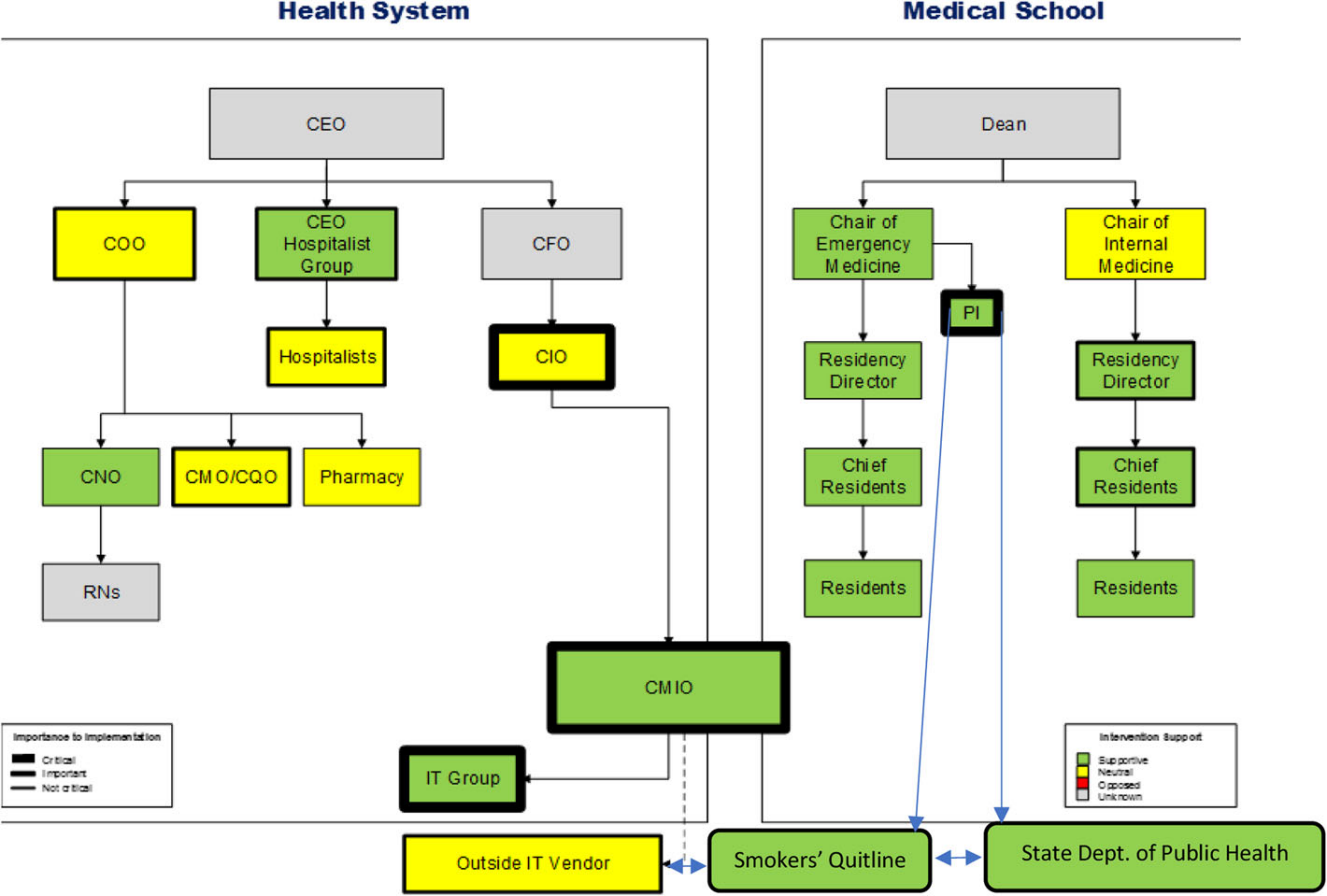
COSMOS visualization of a study using clinical decision support to improve the treatment of hospitalized smokers

Here we provide an illustrative example of a COSMOS from a recent project (Fig. 5). We developed and tested a program of decision support and orders, embedded in an electronic medical record, to improve the identification and treatment of adult smokers admitted to an acute care general hospital in a large academic medical center from 2012 to 2015. Details of the program have been described elsewhere [13]. Reporting standards for this study adhere to the standards for reporting implementation studies (StaRI) statement [14]; a StaRI checklist appears in the Appendix.

In brief, we developed an electronic prompt, linked to an order set, that appeared on the screen the first time an inpatient physician logged on to the chart of a newly admitted patient. If the provider accepted the prompt, three actions were triggered, by default. Each action involves an evidence-based intervention for the treatment of tobacco dependence: (1) an email message was sent to the patient’s primary care provider, informing him/her that tobacco treatment had begun; (2) an electronic referral was sent to the state smokers’ telephone quitline, and the diagnosis of “tobacco use disorder” was added to the patient’s problem list. Physicians could opt out, or unclick, these defaults, if they desired. Third, if the prompt was accepted, the order set appeared next. The order set included a menu of standard medications to treat tobacco dependence; the physician had to click the desired medications. This was the only component of the intervention that required active choice by the physician. All physicians provided written informed consent prior to the study and were randomized to the controls (standard electronic health record features), or the prompt/order set. With a study population of 254 physicians and 1044 patients, we demonstrated statistically significant improvement in the provision of all components of the intervention. However, tobacco cessation rates, assessed via telephone report at 1, 6, and 12 months, and exhaled measurement of breath carbon monoxide at 12 months, did not differ between intervention and controls [15]. The study was approved by the institution’s Human Investigations Committee, and is registered in www.clinicaltrials.gov as NCT01691105.

To design the intervention, the study team spent nearly 2 years developing the COSMOS for the project, displayed in Figure 5. This process consisted of several components: (1) creating an organizational chart for the hospital system; (2) conducting interviews, informal conversations, and email exchanges with individuals identified by study investigators a priori as relevant for the project, including hospital administrators, clinical service chiefs, frontline physicians and nurses, information technology leadership, a pharmacist, and outside vendors and agencies, to assess their level of support and the criticality of that support; (3) snowball sampling [16] to identify other relevant stakeholders; (4) construction of the COSMOS map; and (5) designing and executing actions to secure ongoing support and ensure project success.

We operationalized a stakeholder as anyone who might use the decision support tool (e.g., physicians), design the tool (e.g., information technology personnel, quitline personnel), have to execute orders generated by the tool (e.g., nurses and pharmacists), or provide overall approval for the project (hospital administrators, physician chiefs of clinical services).

### Interpreting the COSMOS

The COSMOS model contains several domains of information:

(1) the formal *organizational chart*, displaying how offices and individuals relate to each other in a formal, systemic structure. This was obtained, in part, from the health system’s website, supplemented by interviews with the chief medical information officer and chief nursing officer. The organizational chart provides the foundation upon which additional detail is layered. It is easily recognized and understood by all members of an implementation team.

(2) the *criticality* of each office or individual to the implementation process, as noted by the thickness of the black margin around the relevant box. The criticality is determined during the formative interviews. Some stakeholders’ criticality may be self-evident: for example, an implementation project that entails changes to the electronic medical record must be supported by the IT leadership team. We chose to trichotomize the level of criticality, in ordinal fashion, for ease of assessment and comprehension. In addition, we think it unlikely that finer gradations of criticality will add useful, actionable information to the implementation team. The three categories of criticality were as follows: (a) *essential*, i.e., the implementation cannot happen without this person’s support; (b) *helpful* to the project, insofar as the individual’s support might facilitate the implementation, but is not essential; and (b) *not essential*, for all other assessments of criticality.

(3) the level of *support* of each of these components for the project. Similar to other models, we wish to denote the degree of support or enthusiasm for the proposed implementation. As with criticality, the COSMOS designates three ordinal levels of support, along with an “unknown” category. Levels of support are assessed through interviews with stakeholders, as well as collateral information obtained from others, insofar as certain stakeholders may, for reasons of social desirability, fail to disclose reservations to the project, or even outright opposition. The three levels of support are as follows: (a) *enthusiastic*, in which the respondent is fully supportive, has no reservations to the implementation, and is likely to champion the program; (b) *supportive,* for respondents who favor the program but may have certain reservations or important competing priorities; and (c) *oppositional*, for respondents who do not support the implementation. (We were aware that some informants might express support, for purposes of social desirability, while remaining opposed to, or unsure about, the implementation.)

In addition to the formal components previously described, important contextual elements may be added to the COSMOS, in the form of observations describing individuals with “soft power” or high centrality (i.e., a dense professional social network) not otherwise mapped, informal relationships between offices and individuals, and any other data relevant to the proposed project. An important caveat is that COSMOS visualizations are institution- and project-specific: implementing identical projects at different institutions may require different models, as will implementing different projects at the same institution. But the COSMOS offers a common map and language to understand the process.

The completed COSMOS provides a quick visual guide to possible project success, facilitators, and barriers. In brief, stakeholders that are critical to project success (thick black outline) and green (supportive) are well-positioned to aid implementation. Any red-colored stakeholder may be in a position to thwart implementation, but in direct proportion to the thickness of the accompanying box outline. These visual cues can assist the implementation team in deciding where to allocate additional resources, or to identify additional approaches to secure support.

## Results

Approximately 2 years of formative work preceded project implementation. This included about 15 months of interviews and planning while writing the grant that ultimately funded the project, and an additional 9 months of work after funding was secured, but concurrent with the onset of implementation. Interviews were conducted with 24 stakeholders, including hospital administrators, information technology leadership, physician leadership from various medical services, resident physicians, and an outside vendor that manages the state’s telephone quitline for smokers (see Table 1). We present this table as a result, since many interviewees were not identified in advance, and hence the table is a reflection of the snowball sampling during the project. The COSMOS itself, developed in iterative fashion, provided a substantial amount of useful, actionable data for our project. These included the following:

- The most supportive stakeholders were also the most critical to project success: the chief medical information officer, the chief of the hospitalist group, and the directors of the residency training programs in internal medicine and emergency medicine.
- There appeared to be no outright opposition. Several individuals and offices appeared neutral. For example, the healthcare system’s chief information officer, while conceptually supportive, was concerned that our work not interfere with the installation of a new electronic health record (EHR) across the system. He asked us to delay our work pending completion of the initial phases of the installation; we did. The system’s chief operating officer expressed similar views, although he did provide a letter of support for the grant application, which ultimately funded this work.
- It became apparent that formal approval of the health system’s chief executive officer was not essential to project success. Because of the highly clinical nature of the implementation, its involvement of health information technology, and its limitation to a select group of physicians, most key stakeholders were either physicians on the faculty of the medical school, or hospitalists employed by the health system, or IT leadership and personnel.
- The realization that the chief information officer (CMIO) reported to the chief financial officer provided a crucial insight that we could frame the intervention as a means to improve the health system’s performance on publicly reported quality measures used by the Centers for Medicare & Medicaid Services in pay-for-performance reimbursement plans.
- Because the CMIO’s job description included managing health IT for both the medical school (the faculty practice) and hospital (largely inpatient and primary care settings), we could consolidate our activities in developing the electronic prompt, order set, and reports in one office.

**Table 1:** Individuals interviewed to develop the COSMOS

**Hospital administrators**	
Chief medical officer	
Chief nursing officer	
Director of nursing, heart and vascular service line	
Director, hospitalist service, hospital 1	
Director, hospitalist service, hospital 2	
**Information technology (IT) team**	
Chief information officer	
Chief medical information officer	
IT programmer/analyst	
**Clinical leadership**	
Chair, Department of Emergency Medicine	
Program Director, Emergency Medicine residency	
Chief residents, Emergency Medicine	
Program Director, Internal Medicine residency (Traditional track)	
Program Director, Internal Medicine residency (Primary Care track)	
Program Director, Internal Medicine (Medicine-Pediatrics track)	
Chief residents, Internal Medicine	
Hospitalists	
**Outside agencies**	
Programmers/analysts, electronic health record company	
Director of Tobacco Control Programs, State Department of Public Health	
Quitline services manager, state smokers’ quitline vendor	

## Discussion

Creating the COSMOS map proved useful in our work. It confirmed a number of a priori assumptions we had of certain stakeholders’ criticality and support, identified additional stakeholders and relationships that would need particular attention, and informed us that certain stakeholders’ support that we had thought critical, was, in fact, not needed. In addition, the lack of outright opposition, which we had not expected, was reassuring. These insights helped focus our teams’ work in executing the implementation.

The duration of the stakeholder analysis deserves comment. For this project, approximately 2 years was spent on stakeholder analysis and developing the COSMOS: 15 months while writing the grant application the funded the work, and an additional 9 months after funding was secured. This largely reflects two factors: (1) the timeline needed to write and submit a grant application to the US National Institutes of Health, await a funding decision, and begin the project and (2) the iterative nature of developing this inaugural COSMOS. Future projects of our group will likely have a shorter timeframe. And implementation projects that are not dependent on securing extramural funding may of course map a COSMOS more quickly. Finally, we should note that any stakeholder analysis, if done properly, can take a substantial period of time. In highly matrixed, complex healthcare organizations, it often does take months to do this work. Insofar as writing the code for, and beta testing, our decision support tools also took months, creating the COSMOS map ultimately did not delay the project. Most of the post-grant development time was expected.

There were several limitations to our approach in developing the COSMOS. First, although there are established tools to assess support and criticality [8, 10, 17], we did not use formal survey instruments to assess stakeholder support for the project. Nevertheless, we used contextual analysis techniques to assess these domains. Second, social desirability response bias may have occurred, with some interviewees being hesitant to express concern or skepticism about the program [18]. We used standard approaches for minimizing this bias, such as interviewing multiple stakeholders to triangulate views, encouraging candor, and probing for details supporting their stated views [19].

The visualization was developed in an iterative fashion, with lessons learned incorporated in subsequent versions. Criticality and support were assessed using methods described previously. Snowball sampling, with confirmation from multiple sources, generally affirmed which stakeholders would be supportive, and how critical they would be to the implementation. Somewhat to our surprise, it became evident that there was no substantial, overt opposition to the implementation. Framing the project as clinically sensible and evidence-based appealed to the clinician stakeholders (physicians, nurses, and pharmacists); addressing the project’s alignment with the regulatory and fiscal obligations of the healthcare system appealed to the non-clinical administrative stakeholders. The most critical set of stakeholders was the information technology group. Their support facilitated that of others.

### Contextual elements

Table 2 lists the contextual elements that provided additional important detail needed to understand the COSMOS, and help us operationalize our implementation. To preserve confidentiality, certain elements that discuss specific individuals have been omitted. These included comments addressing the imminent departure from an organization of a key stakeholder, an informal but important relationship between individuals or offices not easily displayed in the COSMOS, and the presence of a highly supportive champion in the IT group that was otherwise modestly supportive.

**Table 2:** Contextual analysis. Domains adapted from Stange and Glasgow [20]. Table adapted from Bernstein et al. [21]

Domain	Findings	Implications for E-STOPS design and implementation
Relevant theory or participant mental models	Push-pull-capacity model for guideline implementation [22]	Provided conceptual model for study and means of framing E-STOPS for various stakeholders
National, state, local public policy	HITECH act encourages adoption of EHRs; tobacco screening, treatment as early publicly reported core measure	Important “push” factors that facilitated framing of intervention to hospital leadership
Pertinent community norms, resources	Primary care access is modest in local community; care often fragmented between hospital, outpatient providers	Use of health IT/EHR designed to facilitate communication between providers
Healthcare system organization, payment systems, IT, other support systems	IT reports to finance; new EHR installed near planned launch of E-STOPS need to address potential return on investment for tobacco treatment, re: pay-for-performance and public reporting of core measures; compliance with CMS, Joint Commission mandates	Need to address potential return on investment for tobacco treatment, re: pay-for-performance and public reporting of core measures; compliance with CMS, Joint Commission mandates
Practice culture, staffing	Physicians, nurses want to treat tobacco dependence; may have limited skills, knowledge, resources to do so	E-STOPS designed to minimize provider workload, provide choice, but make treatment the default choice.
Patient populations, subgroups	Many adult smokers admitted to hospital; hospitalization as period of enforced abstinence, “teachable moment” for tobacco	E-STOPS limited to inpatient units on medical services, to capitalize on
Relevant historical factors, recent events	Steady decline in prevalence of smoking, but undertreatment still common in healthcare settings; growth of value-based performance models	Used to provide rationale for E-STOPS to physicians, nurses, administrators
Culture, motivations surrounding monitoring, evaluation	Physicians want to treat smokers; some concerns about added workload, role of hospital-based personnel in treating tobacco dependence; concerns about performance assessment	Physicians assured that feedback was confidential, would not be shared with supervisors.

### Alignment of interests

Diverse actors have diverse motivations and interests. It is, therefore, essential for the implementing team to identify these motivations and interests and use that information to provide tailored framing for support of the implementation. The tobacco control intervention we proposed was informed by a substantial body of evidence, from decades’ worth of clinical trials, basic science, and clinical practice guidelines [23]. This was sufficient to persuade physicians and nurses, who are clinically focused stakeholders, to support the implementation. However, other stakeholders required additional persuasion. For the highest–ranking members of the organization (the “C-suite”), we highlighted the importance of external fiscal and regulatory factors to garner support. These included public reporting of quality measures, pay-for-performance programs, and the “meaningful use” provisions of the Health Information Technology for Economic and Clinical Health (HITECH) Act [24, 25]. These approaches proved effective.

### The role of power

To some extent, the COSMOS map de-emphasizes the role of formal organizational or institutional power, which is an integral part of other stakeholder visualizations. There are several reasons for this. For many implementation projects, the formal approval of the C-suite may not be necessary. In the example we provide, we did not ascertain the views of the chief executive officer (CEO) of the hospital system; the chief operating officer seemed, at best, neutral (he provided a letter of support). Similarly, we did not solicit the views of the chief financial officer, to whom the critically important chief information officer was a direct report. Conversely, the CEO of the hospitalist group and chief nursing officer were enthusiastic supporters. But others, closer to the project, were highly supportive—the chief medical information officer, leaders of the relevant residency programs, and others in the IT group. Hence, the holders of “power” most relevant to the project may not necessarily reflect the structural power displayed in the organizational chart.

Table 3 summarizes the abbreviations used in the text.

**Table 3:** List of abbreviations

**Abbreviation**	Meaning
AIDED	Assess/Innovate/Develop/Engage/Devolve
CEO	Chief executive officer
CFIR	Consolidated Framework for Implementation Research
CMIO	Chief medical information officer
CMS	Centers for Medicare & Medicaid Services
EHR	Electronic health record
HITECH Act	Health Information Technology for Economic and Clinical Health Act
IT	Information technology

### Integration with other models of implementation

We wish to emphasize that the COSMOS is not meant to represent a new conceptual model or framework of implementation. It is designed largely to provide a means to visualize the stakeholders involved with or proximate to the implementation, their criticality to the project, their level of enthusiasm for the project, and additional relevant context. The COSMOS may be used in conjunction with—not to replace—other standard models and frameworks used to plan implementation studies that explicitly discuss stakeholders or context, including the CFIR [3] and PARiHS [4].

## Conclusion

The COSMOS represents a potentially useful new approach to stakeholder mapping. It offers information similar to that of existing approaches, including specifying stakeholders, their power over the project, and enthusiasm. The visualization of stakeholders via use of organizational charts enhances these approaches. Consequently, the COSMOS offers users a nuanced view of power via proximity to the project, important details regarding the relationships between and among stakeholders, and an at-a-glance overview of all stakeholders, interrelationships, support, and power. In doing so, it can offer the team responsible for implementation insights about where to devote resources to ensuring programmatic success. Although developed for use in a healthcare setting, it may be adapted to other organizational contexts as well, in nonprofit and for-profit sectors. These organizations may be in healthcare, as discussed here, but also governmental agencies, nonprofit organizations, educational systems and institutions, and industry, may benefit from utilizing COSMOS visualizations in implementing and managing change. We encourage others to explore the utility of creating COSMOS maps to support their efforts in implementation projects in healthcare settings.

## Data Availability

Not applicable.
